# Pulley Blocks v. Inspection Pits

**Published:** 1909-09-18

**Authors:** 


### Motoring Notes.

### PULLEY BLOCKS v. INSPECTION PITS.

It is said that sheep will follow one another even over a precipice. Might not this old adage be applied in a modified form to the human being? Why has the "pit" become such a universal part and parcel of nearly all motor houses? The idea is doubtlessly an adaptation borrowed from the "loco. shed," where there is every justification for its presence. Locomotives are too heavy to be lifted by hand even head high if it can be avoided; but with even heavy weights the engineer will for any prolonged work sling or jack his engine up rather than burrow underground like a rabbit. I must confess that the idea of any alternative to the regulation pit never entered my mind until some months ago, when a correspondent in one of the motor journals made a most valuable and interesting suggestion whereby inspection pits, with their manifold disadvantages, could be dispensed with. These pits are often difficult to construct, as drainage in most cases must be provided for. They are dangerous, as the list of accidents proves, owing to accumulation of vapour. They harbour dirt, dust, and damp, and when not covered over are difficult to negotiate when bringing a car in, especially at night. Lastly, every time a fresh tool is required a more or less acrobatic feat has to be gone through before it can be reached. Few cars scale more than 30 cwt., the doctor's, as a rule, not more than 15 cwt., and the alternative suggested is pulley blocks.

Providing the motor house is one converted out of an old coach house or stable, with brick or stone walls, get a piece of timber, pitch pine or oak for choice, and place across the house, lodging each end on the wall plate. The ends may be sloped off to let it get well over, if necessary, or a brick bracket can be built as an extra support; failing this a 4½-inch pier could be built from the floor, or a 9-inch by 3-inch deal placed on end and bolted to the wall. If the shed sides are only wood, a 9-inch brick pier would be necessary, or a piece of timber as stated braced to the side.

The size of the cross-piece must depend on the width of span, but any builder would state what was necessary. In the centre of the stretcher put a clip or iron band with a ring suspended therefrom; from this the hook of the pulley blocks would hang; a pair of Weston blocks will suspend a car at any height without special provision. Pass two rope slings under the chassis fore and aft, and raise the car to any height you want. The adjustments, alterations, or repairs can be done in sight, and no lamps, with consequent dangers, are required.

There are few motor houses but what have headroom enough to elevate a car. To prevent the slings closing in too quickly a wooden stretcher can be used. The foregoing is, of course, only intended to apply to private garages and not to trade ones.

The approximate cost of apparatus sufficient to lift cars of at least 30 cwt. would be:

| | | | |
|---|---|---|---|
| One set of Weston's self-holding pulley blocks and chains ... ... ... ... ... | £2 | 0 | 0 |
| Two rope slings ... ... ... ... ... | 1 | 0 | 0 |
| Timber or brickwork and cross piece ... ... | 3 | 0 | 0 |
| | £6 | 0 | 0 |

With the ropes so placed that the weight is about equal, there is not the least fear of springing the chassis. The author of the arrangement says he generally removes the body first with the aid of the lifting apparatus, and then slings the chassis.

